# Undiagnosed dementia and mortality among older adults in the United States and Brazil: A cross‐national cohort study

**DOI:** 10.1002/alz.71430

**Published:** 2026-04-29

**Authors:** Márlon Juliano Romero Aliberti, Thiago J. Avelino‐Silva, Kenneth E. Covinsky, Kenneth Lam, Sei J. Lee, Laiss Bertola, Cleusa P. Ferri, Maria Fernanda Lima‐Costa, Juliana Vaz de Melo Mambrini, Andrew Christopher Claro Miguel, Alden L. Gross, Claudia Kimie Suemoto

**Affiliations:** ^1^ Laboratorio de Investigacao Medica em Envelhecimento (LIM‐66) Servico de Geriatria Hospital das Clinicas HCFMUSP, Faculdade de Medicina, Universidade de Sao Paulo Sao Paulo Brazil; ^2^ Research Institute Hospital Sirio‐Libanes São Paulo Brazil; ^3^ Department of Epidemiology Johns Hopkins Bloomberg School of Public Health, Johns Hopkins University Baltimore Maryland USA; ^4^ Division of Geriatrics Department of Medicine University of California, San Francisco San Francisco California USA; ^5^ Division of Geriatric Medicine Department of Medicine University of Colorado Anschutz Medical Campus Aurora Colorado USA; ^6^ Department of Psychiatry, Escola Paulista de Medicina, Departamento de Psiquiatria Universidade Federal de São Paulo São Paulo Brazil; ^7^ Núcleo de Estudo em Saúde Pública e Envelhecimento (NESPE) da Universidade Federal de Minas Geraise da Fundação Oswaldo Cruz Belo Horizonte Minas Gerais Brazil

**Keywords:** cross‐national studies, dementia, diagnosis, health equity, mortality, older adults, prognosis, undiagnosed dementia

## Abstract

**INTRODUCTION:**

Cross‐national evidence on undiagnosed dementia and its prognostic implications remains limited. We compared the proportion of undiagnosed dementia, associated factors, and mortality in the United States and Brazil.

**METHODS:**

This population‐based cohort study included adults aged ≥ 65 years from the 2016 US Health and Retirement Study (*n* = 9,539) and the 2015–2016 Brazilian Longitudinal Study of Aging (*n* = 3603), followed for mortality through 2020. Dementia was classified as no dementia, undiagnosed dementia, or diagnosed dementia using harmonized cognitive, functional, and informant measures.

**RESULTS:**

The proportion of undiagnosed dementia was higher in Brazil (76.1%) than in the United States (45.1%). Undiagnosed dementia was associated with increased 4‐year mortality compared with no dementia in both countries. It was linked to younger age and absence of memory complaints, with marked socioeconomic and healthcare disparities in Brazil.

**DISCUSSION:**

Undiagnosed dementia is common and associated with increased mortality, identifying a vulnerable population missed by current diagnostic pathways.

## Highlights

- About half of dementia cases are undiagnosed in the United States and three‐quarters in Brazil
- Undiagnosed dementia was associated with higher mortality risk in the United States and Brazil
- Younger age and absence of memory complaints were linked to undiagnosed dementia
- The diagnostic gap was marked by socioeconomic and healthcare disparities in Brazil
- Undiagnosed dementia identified a vulnerable population missed by health systems

## Graphical abstract

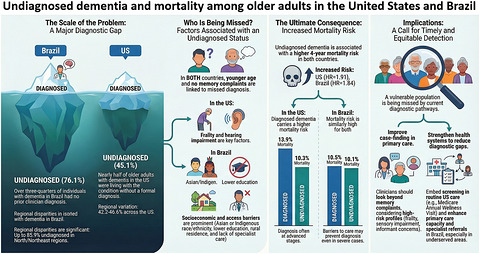

## 1 BACKGROUND

Dementia is a major and growing global challenge in aging societies, affecting an estimated 57 million people worldwide, with the fastest growth occurring in low and middle‐income countries (LMICs).^1^ As populations age, timely identification of older adults at risk becomes increasingly important to improve care and reduce avoidable harms. Delayed dementia diagnosis has practical consequences because patients and families may miss opportunities for counseling, safety planning, medication review, and caregiver engagement in managing comorbid illness across care settings.^2^

A measurable and potentially modifiable gap is the proportion of individuals who meet criteria for dementia but have not received a clinician diagnosis.^3^ In the United States (US), prior work suggests that nearly 40% of older adults meeting dementia criteria lack a documented diagnosis.^4^ In lower‐resource settings, where access to specialists and diagnostic infrastructure may be limited, this gap is likely larger, reaching up to 80% in settings such as Brazil.^5^, ^6^ Fragmented health systems, limited provider training, and low public awareness may further delay recognition and exacerbate inequities in care.^7^ At the same time, undiagnosed dementia may also reflect differences in disclosure practices, patient preferences, and reporting practices, which vary across health systems and cultures.^2^, ^8^

Although universal cognitive screening remains controversial, targeted case‐finding approaches have been proposed as more efficient and equitable strategies for identifying older adults at the highest risk of dementia.^9^ However, most existing evidence on undiagnosed dementia comes from single‐country studies, largely in high‐income settings, and cross‐national comparisons using harmonized dementia ascertainment are uncommon.^10^ Moreover, the prognostic implications of undiagnosed dementia across health systems with differing socioeconomic and structural characteristics remain uncertain.

Using nationally representative cohorts of community‐dwelling older adults in the United States and Brazil, we estimated the proportion of dementia cases without a reported prior diagnosis, identified sociodemographic, clinical, and health system factors associated with diagnostic gaps, and examined the association between undiagnosed dementia and 4‐year mortality.

## 2 METHODS

### 2.1 Study design and population

This longitudinal observational study used cross‐national data from two population‐based cohorts, including the US Health and Retirement Study (HRS) and the Brazilian Longitudinal Study of Aging (ELSI‐Brazil), to examine undiagnosed dementia among community‐dwelling adults aged 65 years and older in the United States and Brazil.^11^, ^12^ Both cohorts are part of the family of international aging studies derived from the HRS and include comparable measures harmonized through the Gateway to Global Aging Data.^13^ Detailed cohort methods have been published elsewhere.^11^, ^12^

**RESEARCH IN CONTEXT**

- **Systematic review**: We searched PubMed, Scopus, and Web of Science for population‐based studies on undiagnosed dementia, its determinants, and outcomes. Prior studies, mainly from high‐income countries, reported that 40‐60% of dementia cases are undiagnosed, with higher proportions in low‐ and middle‐income countries. Population‐based evidence linking undiagnosed dementia to mortality is limited, and cross‐national comparisons using harmonized methods remain scarce.
- **Interpretation**: Using nationally representative cohorts, we showed that undiagnosed dementia is common in high‐ and lower‐income settings, with a higher burden in Brazil than in the United States and marked socioeconomic and healthcare disparities. Undiagnosed dementia was associated with increased 4‐year mortality in both countries, identifying a clinically vulnerable population not reached by current diagnostic pathways.
- **Future directions**: Future studies should examine patient‐centered outcomes, including functional trajectories and quality of life, and assess whether improving dementia awareness, diagnosis, and access to care reduces mortality risk and health inequities across health systems.

HRS is a nationally representative biennial survey of US adults aged 50 years and older.^11^ We used the 2016 wave (Wave 13), which included direct cognitive testing and physical performance measures for participants aged 65 years and older living in the community. ELSI‐Brazil is a nationally representative cohort of community‐dwelling adults aged 50 years and older residing in 70 municipalities across all five Brazilian macro‐regions; baseline data were collected in 2015–2016 via home interviews.^12^

We restricted both cohorts to participants aged 65 years and older because detailed cognitive assessments in HRS were administered only from age 65 years onward. For comparability, we aligned baseline timing using HRS 2016 and ELSI‐Brazil 2015–2016. All 9,993 HRS participants aged 65 years and older and all 3,860 ELSI‐Brazil participants aged 65 years and older were eligible for the study. We excluded participants with missing cognitive measures (244 in the United States and 141 in Brazil) or covariate data (210 in the United States and 116 in Brazil). The final sample included 9,539 HRS participants and 3,603 ELSI‐Brazil participants (Figure **S1**).

HRS and ELSI‐Brazil received ethical approval from the University of Michigan and the Oswaldo Cruz Foundation, respectively. This study is reported in accordance with the Strengthening the Reporting of Observational Studies in Epidemiology (STROBE) reporting guideline, as detailed in the Supplement.

### 2.2 Undiagnosed dementia

In both studies, we defined undiagnosed dementia as dementia‐level cognitive impairment identified by objective cognitive testing (self‐respondents) or informant report (proxy‐respondents), in the absence of self‐ or proxy‐report of a clinician diagnosis of Alzheimer's disease or dementia.^14^, ^15^ We ascertained evidence of dementia using either (A) cognitive impairment with functional impairment or (B) reported clinician diagnosis, and classified dementia as diagnosed (B present) or undiagnosed (A present without B) (**Figure** **S2**).

Evidence for dementia based on criterion A differed by respondent type. Among self‐respondents, cognitive status was assessed across four domains: memory (immediate and delayed 10‐word recall), orientation (day of week, day, month, year), verbal fluency (animal naming), and language (two object‐naming items and two items on general knowledge).^14^ Cognitive impairment was defined using domain‐specific z scores as performance ≤ −1.5 standard deviation (SD) below the normative mean in at least two domains, or ≤ −1.5 SD in one domain plus ≤ −1.0 SD in at least two additional domains.^15^, ^16^ Normative means and SDs were estimated separately within each cohort using an internal subcohort with characteristics consistent with normal cognition (e.g., no major comorbidities or functional limitations). We then computed each participant's expected normative test performance based on age, sex, and education. Criterion A additionally required functional impairment, defined as difficulty or inability to perform at least one basic activity of daily living (ADL: dressing, toileting, bathing, eating, transferring, or walking across a room) or at least two instrumental activities of daily living (IADL: managing money, using the telephone, taking medications, shopping, preparing meals, or housekeeping) without assistance.^15^, ^16^, ^17^ Among proxy‐respondents (n = 730 [5.5%]), dementia was assessed using the 16‐item Informant Questionnaire on Cognitive Decline in the Elderly (IQCODE‐16); scores ≥ 3.4 indicated dementia.^6^

### 2.3 Factors associated with undiagnosed dementia

We examined factors associated with undiagnosed dementia, including sociodemographic characteristics, comorbidities, geriatric conditions, and healthcare access and utilization.^10^, ^18^, ^19^ Variables were selected a priori based on prior literature, and definitions were harmonized across cohorts.

**Sociodemographic characteristics**. Variables included age (continuous), sex (male/female), and race/ethnicity (White, Black, other). In HRS, other included Asian, American Indian/Alaska Native, Native Hawaiian/Pacific Islander, Hispanic ethnicity (regardless of race), and multiracial. In ELSI‐Brazil, other included Asian or Indigenous (Native Brazilians). Individuals identifying as Black or of mixed Black and White ancestry in Brazil were classified as Black to reflect similar socioeconomic patterns and health outcomes.^20^ Additional variables included years of education, household income, marital status (married/partnered vs. other), residence (urban vs. rural), and geographic region. Education and income were categorized within each country using the lowest quartile as the threshold for lower educational attainment and lower income. In the United States, lower educational attainment was ≤ 11 years, and lower income was ≤ 28,800 US dollars (USD)/year; in Brazil, lower educational attainment was no formal schooling, and lower income was ≤ 5,600 USD/year. Regions were categorized to reflect structural inequalities in the United States (Northeast, Midwest, South, West) and in Brazil (North/Northeast, Midwest, South/Southeast).^21^, ^22^

**Health comorbidities**. Chronic diseases, including hypertension, diabetes, cancer, lung disease, stroke, osteoarthritis, heart disease, and depression, were assessed through self‐ or proxy‐report of a previous physician diagnosis. Multimorbidity was defined as two or more conditions.^23^

**Geriatric conditions**. We included memory complaints, self‐rated health, frailty, and sensory impairment. Memory complaints were defined as rating one's memory as fair/poor (vs. excellent/very good/good) or reporting worsening memory over the past 2 years. Self‐rated health was defined as fair/poor (vs. excellent/very good/good). Frailty was defined using the FRAIL scale (fatigue, resistance, ambulation, illness, weight loss), with frailty defined as three or more criteria.^24^ Hearing and vision impairment were defined as self‐rated fair/poor (vs. excellent/very good/good). Proxy respondents provided ratings when participants were unable to self‐report.

**Healthcare access and utilization**. Variables included usual source of care, specialized care, recent hospitalization, and out‐of‐pocket expenditures, each harmonized and coded as binary indicators (yes/no) to ensure comparability across cohorts.^8^

### 2.4 Mortality data

Mortality over 4 years (2016–2020) was ascertained using cohort‐specific sources. In HRS, deaths were identified through linkage to the National Death Index and Medicare claims, supplemented by exit interviews with family proxies. In ELSI‐Brazil, deaths were identified using proxy informant reports and probabilistic linkage to the national Mortality Information System (SIM). These sources provided all‐cause mortality status and date of death in both countries. Participants with no death recorded during follow‐up were censored at the end of the 4‐year follow‐up.

### 2.5 Statistical analysis

Details of the normative subcohort selection and standardization procedures, adapted from prior work, are provided in the Supplement (Supplementary Methods).^14^, ^15^ The normative subcohort included 2099 participants in HRS (22.0%) and 787 in ELSI‐Brazil (21.8%). Characteristics of normative and non‐normative participants are shown in the Table S1.

All analyses accounted for the complex survey design and sample weights to generate population‐based estimates. We calculated the proportion of participants with undiagnosed dementia in the United States and Brazil overall and by region. To enable fair cross‐national comparisons of dementia prevalence, we computed age‐ and sex‐adjusted prevalence estimates using marginal standardization. Baseline characteristics were presented by dementia classification status (no dementia, undiagnosed dementia, and diagnosed dementia) and compared between undiagnosed and diagnosed dementia using survey‐adjusted chi‐square tests for categorical variables and survey‐weighted t‐tests or Wilcoxon rank‐sum tests for continuous variables, as appropriate.

To identify factors associated with undiagnosed dementia, we fit multivariable Poisson regression models comparing undiagnosed with diagnosed dementia within each country, including sociodemographic characteristics, multimorbidity, geriatric conditions, and healthcare access and utilization. Results were expressed as prevalence rate ratios (PRRs) with 95% confidence intervals (CIs).

To examine the association between undiagnosed dementia and mortality, we estimated age‐ and sex‐adjusted standardized cumulative mortality curves over up to 4 years of follow‐up by dementia group within each country. We then fit Cox proportional hazards models to estimate hazard ratios (HRs) for undiagnosed dementia, using no dementia as the reference group, in two models: (1) adjusted for sociodemographic characteristics and (2) additionally adjusted for clinical measures (multimorbidity, frailty, and recent hospitalization). We also estimated HRs comparing undiagnosed dementia with diagnosed dementia. We tested an interaction between undiagnosed dementia and cohorts to assess whether associations with 4‐year mortality differed between countries. In a sensitivity analysis, we restricted the sample to self‐respondents to evaluate whether proxy‐assessed cognitive impairment, as measured by the IQCODE‐16, affected the mortality associations.

We performed a complete‐case analysis due to a low missing data rate (5.1%).^25^ Analyses were conducted using Stata 17 (StataCorp LLC, College Station, TX, USA), with two‐sided tests and a significance level of 0.05.

## 3 RESULTS

### 3.1 Study population

The sample comprised 9539 participants from the United States and 3603 from Brazil. In the US cohort, participants were older (mean age [SD], 74.6 [7.8] vs. 73.4 [6.9] years), more often identified as White (85.8% vs. 43.1%), and had higher educational attainment (median, 12 vs. 3 years) and annual household income (median, 47 vs. 8,000 USD) than participants in the Brazilian cohort (**Table** **S2**). US participants also reported a greater burden of chronic conditions and higher health care access and use, including having a usual source of care, specialty service use, and higher out‐of‐pocket health care spending.

### 3.2 Prevalence and associated factors of undiagnosed dementia

**FIGURE 1:**
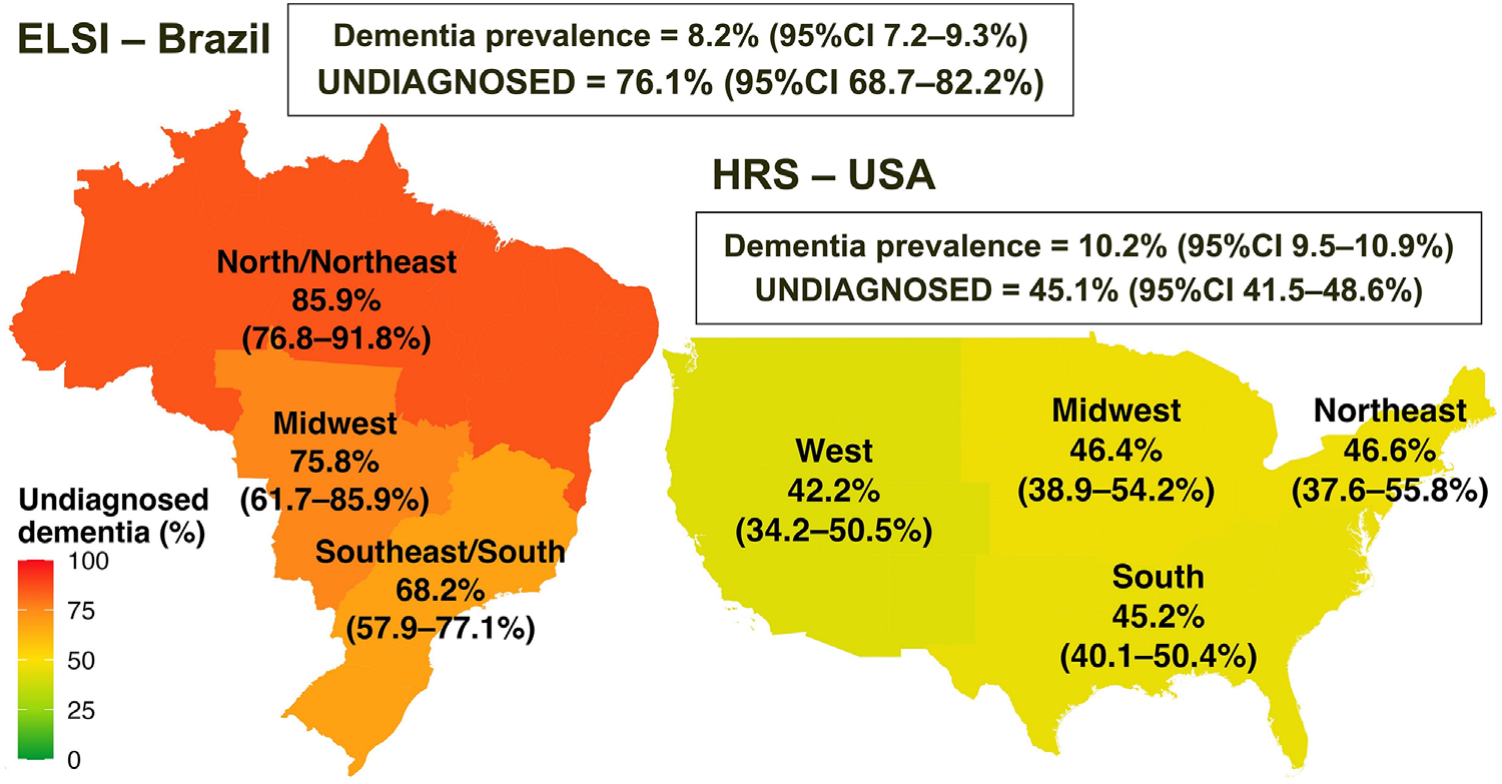
Proportion of dementia that is undiagnosed in the United States and Brazil, overall and by region. Regional colors follow a continuous heatmap scale, with darker tones representing higher proportions of undiagnosed dementia. All percentage estimates were survey‐weighted to represent the older population in each country and are shown with their 95% CI. 95% CI, 95% confidence interval.

In the United States, 8,410 participants were classified as having no dementia, 494 as having undiagnosed dementia, and 635 as having diagnosed dementia; in Brazil, 3,271, 261, and 71 participants, respectively (Table 1). The prevalence of study‐defined dementia was 10.2% in the United States and 8.2% in Brazil. After adjustment for age and sex, prevalence estimates were nearly identical between countries (7.9% [95% CI = 7.3%–8.5%] in the United States vs. 8.0% [95% CI = 6.9%–9.0%] in Brazil). Although the proportion of undiagnosed dementia was high in both countries, it was markedly higher in Brazil (76.1%) compared to the United States (45.1%), as detailed in Figure 1. Within‐country variation was more pronounced in Brazil, where the proportion of undiagnosed dementia reached 85.9% in the North/Northeast and 68.2% in the Southeast/South, compared with smaller regional differences in the United States (42.2%‐46.6%).

**TABLE 1:** Characteristics by dementia status among older adults in the United States and Brazil

	Dementia (HRS – United States)				Dementia (ELSI‐Brazil)			
	Absent	Undiagnosed	Diagnosed		Absent	Undiagnosed	Diagnosed	
Variables	**(*n* = 8410)**	**(*n* = 494)**	**(*n* = 635)**	*p‐*Value^a^	**(*n* = 3271)**	**(*n* = 261)**	**(*n* = 71)**	*p‐*Value^a^
Cognitive and functional measures								
Orientation (0‐4), mean (SD)	3.7 (0.6)	2.8 (1.1)	2.9 (1.2)	0.27	3.4 (1.0)	1.5 (1.1)	2.1 (1.5)	0.06
Immediate recall (0‐10), mean (SD)	5.3 (1.6)	3.2 (1.5)	3.3 (1.8)	0.47	3.8 (1.6)	2.2 (1.5)	2.7 (1.3)	0.02
Delayed recall (0‐10), mean (SD)	4.4 (1.9)	2.0 (1.7)	2.0 (1.9)	0.95	2.3 (1.8)	0.7 (1.1)	0.6 (0.8)	0.72
Verbal fluency, mean (SD)	17.4 (6.6)	10.3 (5.8)	10.4 (6.1)	0.88	11.2 (4.0)	6.6 (4.0)	7.9 (3.2)	0.02
Language (0‐4), mean (SD)	3.3 (1.2)	1.7 (1.1)	2.4 (1.2)	<0.001	2.7 (0.9)	1.6 (0.8)	2.3 (1.0)	0.002
IQCODE‐16, mean (SD)^b^	2.0 (0.6)	4.2 (0.4)	4.6 (0.6)	<0.001	2.4 (0.5)	4.3 (0.5)	4.3 (0.9)	0.68
ADL limitations (0‐6), mean (SD)	0.3 (0.9)	1.9 (1.9)	2.8 (2.4)	<0.001	0.4 (1.1)	2.1 (2.1)	2.8 (2.5)	0.09
IADL limitations (0‐6), mean (SD)	0.3 (1.0)	2.3 (1.9)	4.0 (2.2)	<0.001	0.8 (1.4)	3.5 (1.9)	3.8 (2.1)	0.34
Sociodemographic characteristics								
Age (years), mean (SD)	74.0 (7.3)	78.1 (8.9)	82.2 (8.4)	<0.001	73.0 (6.7)	77.0 (8.5)	80.1 (7.2)	0.02
Female sex, %	55.8	56.4	62.2	0.11	56.5	67.4	68.9	0.86
White race/ethnicity, %				0.32				0.09
White	86.5	79.1	80.8		43.7	32.8	43.1	
Black	8.8	13.4	14.3		48.8	56.9	54.3	
Other	4.7	7.5	4.9		7.5	10.3	2.5	
Education (years), median (IQI)	12 (12, 16)	12 (11, 14)	12 (10, 14)	0.13	4 (1, 5)	1 (0, 4)	3 (0, 4)	0.001
Income (in 1,000 USD), median (IQI)^c^	49 (30, 87)	33 (21, 55)	37 (24, 61)	0.003	8 (6, 13)	7 (5, 10)	8 (6, 13)	0.002
Married or partnered, %	58.3	45.5	36.1	0.009	55.0	38.3	47.4	0.33
Rural living, %	28.4	33.4	27.1	0.07	16.4	22.2	8.8	0.03
Health comorbidities								
Hypertension, %	65.5	79.0	77.7	0.67	64.5	59.2	64.3	0.47
Diabetes, %	26.8	36.2	31.5	0.17	18.6	25.6	26.9	0.86
Cancer, %	20.1	22.3	23.2	0.74	8.2	4.5	11.8	0.09
Lung disease, %	10.8	22.8	17.2	0.05	6.1	7.7	13.0	0.17
Stroke, %	8.9	27.3	34.4	0.04	6.8	15.6	27.9	0.06
Osteoarthritis, %	68.6	76.7	77.2	0.86	24.0	26.6	30.9	0.58
Heart disease, %	12.6	23.3	23.2	0.96	16.5	13.0	25.2	0.02
Depression, %	20.7	33.0	48.9	<0.001	15.4	19.7	41.7	0.001
Multimorbidity (≥ 2 LTCs), %	71.8	85.7	87.4	0.51	48.4	54.0	67.9	0.06
Geriatric conditions								
Memory complaints, %	39.6	60.9	81.3	<0.001	53.2	77.1	89.1	0.06
Self‐rated poor health, %	24.1	58.5	64.1	0.11	11.4	20.6	27.5	0.33
Frail status, %	13.3	46.1	42.8	0.37	13.5	42.1	46.6	0.59
Hearing impairment, %	22.7	42.0	43.2	0.73	25.5	42.6	45.3	0.72
Visual impairment, %	20.8	40.7	51.6	0.003	21.2	38.6	32.9	0.45
Healthcare access and utilization								
Having a usual provider, %	90.1	84.8	87.3	0.32	65.0	73.0	74.7	0.81
Received specialist care, %	74.2	65.1	73.0	0.02	57.5	47.4	69.5	0.002
Recent hospitalization, %	26.8	48.1	55.4	0.04	11.4	16.0	24.4	0.14
Out‐of‐pocket expenditure, %	88.7	78.9	81.2	0.45	29.4	29.0	32.2	0.66

*Note*: All estimates are survey‐weighted and account for the complex sampling design of the Health and Retirement Study (HRS) in the United States and the Brazilian Longitudinal Study of Aging (ELSI‐Brazil).

^a^ *p*‐Values represent comparisons between undiagnosed and diagnosed dementia within each country; the no‐dementia group is shown for descriptive context.

^b^ IQCODE‐16 = 16‐item Informant Questionnaire on Cognitive Decline in the Elderly (performed by 503 proxies in the United States and 227 in Brazil due to participants’ inability to complete cognitive testing).

^c^ Annual household income reported in thousands of US dollars (1,000 USD).

Abbreviations: ADL limitations, difficulty performing basic activities of daily living (dressing, toileting, bathing, eating, transfers, or walking); IADL limitations, difficulty performing instrumental activities of daily living (managing money, using the telephone, taking medications, shopping, preparing meals, or housekeeping); IQI, interquartile interval; LTC, long‐term conditions; SD, standard deviation; USD, United States dollar.

Participants with undiagnosed dementia had worse cognitive and functional performance than those without dementia in both countries (Table 1). Compared with diagnosed dementia, undiagnosed dementia showed broadly similar cognitive performance, with lower language scores in both countries; functional limitations were lower in the United States but similar in Brazil. Depression diagnosis was less common in undiagnosed than diagnosed dementia (Table 1).

**FIGURE 2:**
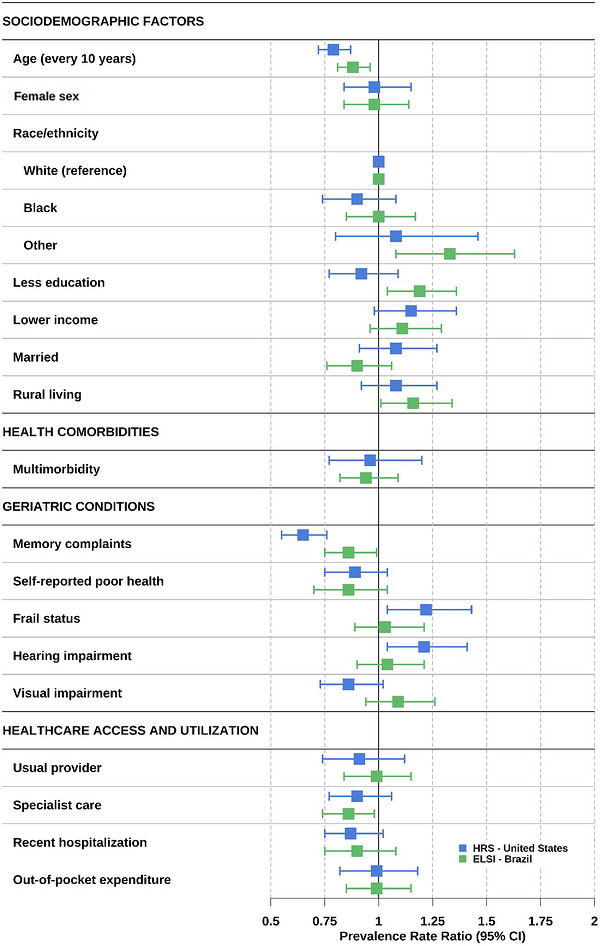
Factors associated with undiagnosed versus diagnosed dementia among older adults in the United States and Brazil. Estimates were computed from multivariable survey‐weighted Poisson regression models comparing undiagnosed dementia to diagnosed dementia, adjusting for all listed factors. Reference groups: male sex (for female sex), White (for race/ethnicity), higher educational attainment and income level (for less education and lower income levels), without partner (for married), urban area (for rural), and absence of multimorbidity, memory complaints, poor health, frailty, hearing or visual impairment, usual provider, specialist care, recent hospitalization, or out‐of‐pocket expenditure. Educational attainment and annual household income were classified within each country as the lowest quartile (≤ 11 years of schooling or ≤ 28,800 USD in the United States; no formal education or ≤ 5,600 USD in Brazil). 95% CI, 95% confidence interval; USD, United States dollars.

In multivariable analysis, among participants with dementia, undiagnosed dementia was associated with younger age and absence of memory complaints in both countries (Figure 2). In the United States, frailty and hearing impairment were also associated with undiagnosed dementia. In Brazil, undiagnosed dementia was associated with Asian or Indigenous race/ethnicity, fewer years of education, and rural residence; lower specialist care use was also associated with undiagnosed dementia in Brazil (Figure 2).

### 3.3 Mortality

**FIGURE 3:**
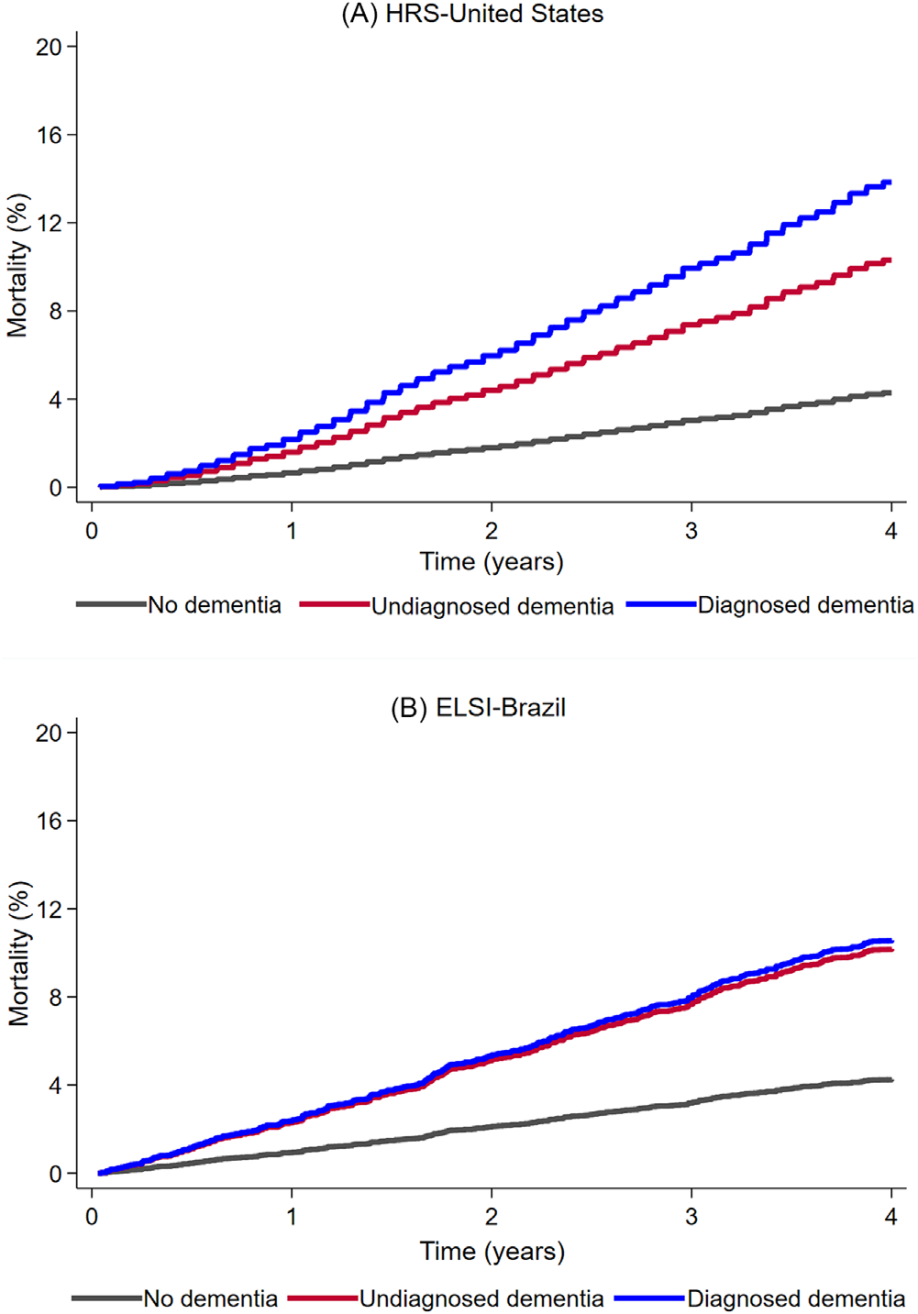
Age‐ and sex‐adjusted cumulative mortality by dementia status over 4 years in the United States and Brazil. Age‐ and sex‐adjusted cumulative mortality was higher for diagnosed than undiagnosed dementia in the United States (*p* = 0.003) but did not differ in Brazil (*p* = 0.89). HRS, Health and Retirement Study; ELSI‐Brazil, Brazilian Longitudinal Study of Aging.

Median follow‐up was 4 years, and 96.5% of participants had complete follow‐up through death or 4 years. Age‐ and sex‐adjusted standardized 4‐year cumulative mortality curves showed higher mortality among participants with undiagnosed or diagnosed dementia than among those without dementia in the United States and Brazil (Figure 3). In the United States, age‐ and sex‐adjusted 4‐year cumulative mortality was lower for undiagnosed than diagnosed dementia (10.3% vs. 13.9%; *P* = 0.003), whereas mortality did not differ in Brazil (10.1% vs. 10.5%; p = 0.89). After adjustment for sociodemographic and clinical factors, both undiagnosed and diagnosed dementia was associated with higher 4‐year mortality compared with no dementia in both countries (Table 2). Compared with diagnosed dementia, undiagnosed dementia was associated with lower mortality in the United States (HR = 0.75; 95% CI = 0.61–0.92) but not in Brazil (HR = 0.89; 95% CI = 0.50–1.59). There was no evidence of effect heterogeneity by country (interaction HR = 0.92; 95% CI = 0.63–1.33). In a sensitivity analysis restricted to self‐respondents, undiagnosed dementia remained associated with higher 4‐year mortality compared with no dementia in both countries (Table S3).

**TABLE 2:** Association of dementia status with 4‐year mortality among older adults in the United States and Brazil

Parameter	Hazard ratio (95% confidence interval)			
	Model 1: Adjusted for sociodemographic factors	Model 1: Undiagnosed vs. diagnosed dementia	Model 2: adjusted for sociodemographic and clinical factors.	Model 2: Undiagnosed vs. diagnosed dementia
HRS‐United States (n = 9539)				
No dementia	(reference)		(reference)	
Undiagnosed dementia	2.40 (2.00–2.89)	(reference)	1.91 (1.59–2.30)	(reference)
Diagnosed dementia	3.29 (2.82–3.83)	0.73 (0.60–0.90)	2.55 (2.18–2.99)	0.75 (0.61–0.92)
ELSI‐Brazil (n = 3603)				
No dementia	(reference)		(reference)	
Undiagnosed dementia	2.24 (1.62–3.12)	(reference)	1.84 (1.32–2.57)	(reference)
Diagnosed dementia	2.66 (1.56–4.54)	0.84 (0.47–1.53)	2.07 (1.23–3.49)	0.89 (0.50–1.59)

*Note*: Estimates were derived from Cox proportional hazards models examining the association between dementia status and time to death over a 4‐year period. Two models were fitted: Model 1, adjusted for sociodemographic characteristics (age, sex, race/ethnicity, education, income, marital status, and place of residence); Model 2, adjusted for sociodemographic characteristics and clinical measures (multimorbidity, frailty, and recent hospitalization).

Abbreviations: ELSI‐Brazil,  Brazilian Longitudinal Study of Aging; HRS, United States Health and Retirement Study.

## 4 DISCUSSION

This cross‐national cohort study indicates that undiagnosed dementia is a substantial public health problem among older adults in the United States and Brazil. Nearly half of older adults with dementia in the United States and more than three‐quarters in Brazil did not report receiving a formal diagnosis, exposing major gaps in dementia detection across distinct socioeconomic contexts. Undiagnosed dementia was associated with younger age and absence of memory complaints in both countries. In the United States, individuals with undiagnosed dementia had less functional impairment than those with diagnosed dementia. In Brazil, undiagnosed dementia was more common among members of underrepresented racial and ethnic groups, those with lower educational attainment, and those living in rural areas. Undiagnosed dementia was associated with higher 4‐year mortality than no dementia in the United States and with a mortality risk similar to diagnosed dementia in Brazil.

Few cross‐national studies using comparable methods have examined undiagnosed dementia across high‐ and lower‐income settings. Prior population‐based studies, mainly from Europe and North America, suggest that approximately 40% to 65% of dementia cases are undiagnosed.^10^ Estimates from LMICs have been higher, including reports from China and India, in which 70% to 90% of cases were unrecognized, consistent with our findings in Brazil.^6^, ^10^, ^15^ To better contextualize cross‐national differences, we also examined overall dementia prevalence and found that age‐ and sex‐adjusted estimates were similar between countries, suggesting that differences in crude prevalence were largely driven by population composition rather than underlying disease burden. Similar to our US findings, prior studies in high‐income settings have linked undiagnosed dementia to milder cognitive and functional impairment.^4^, ^14^ Other work has also linked social isolation, which often co‐occurs with frailty and hearing impairment, to undiagnosed dementia.^26^ In LMICs, socioeconomic disadvantage, geographic barriers, and limited access to specialized health care services may play an important role, as observed in Brazil.^27^

In addition to structural barriers, cultural‐ and communication‐related factors may contribute to the high proportion of undiagnosed dementia observed in Brazil.^28^ Although these processes are present in high‐income settings such as the United States, they appear more pronounced in Brazil due to lower dementia literacy and less structured diagnostic pathways. For example, cognitive decline may be interpreted as normal aging in both countries, delaying recognition and help‐seeking; however, this perception is more prevalent in Brazil, particularly among populations with lower educational attainment.^29^, ^30^ Clinicians may also attribute early symptoms to aging in both settings; in Brazil, limited training, absence of standardized pathways, and reduced access to specialists may further amplify diagnostic delays.^31^, ^32^ While stigma influences disclosure in both countries, selective disclosure within families and the use of indirect language are particularly well documented in Brazil.^33^, ^34^ Family compensation for cognitive deficits may also obscure functional decline, though its interaction with healthcare access may differ across settings. Variability in diagnostic communication may influence awareness; yet self‐ or proxy‐reported diagnoses may underestimate clinical recognition more frequently in Brazil, where disclosure practices are less standardized.^4^, ^35^ Together, these factors indicate that the high burden of undiagnosed dementia reflects an interplay between healthcare access and culturally shaped perceptions of aging and disease, with a greater impact in lower‐resource settings.^8^, ^21^, ^29^

Undiagnosed dementia identified a group of older adults at increased risk of death in the United States and Brazil. To our knowledge, this is the first population‐based, cross‐national study to demonstrate the prognostic relevance of undiagnosed dementia for mortality across settings using nationally representative cohorts of older adults. In the United States, higher mortality among those with diagnosed dementia than among those with undiagnosed dementia is consistent with diagnoses occurring at more advanced stages, when cognitive and functional impairment and overall clinical vulnerability are greater.^4^ In Brazil, the near overlap of mortality curves for undiagnosed and diagnosed dementia suggests that diagnostic status may be less tightly aligned with disease severity.^6^ Barriers to specialized care may delay or prevent formal diagnosis even among older adults with clinically significant dementia.^7^ Alternatively, diagnosis may be more closely linked to access to resources and continuity of care, which may mitigate mortality risk and narrow differences between groups.^8^, ^36^ These findings suggest that estimates based only on diagnosed dementia may underestimate dementia‐related mortality.

Our finding that undiagnosed dementia was associated with increased mortality identifies missed identification in routine care as a marker of higher clinical risk, supporting the potential value of targeted case‐finding approaches to identify individuals at increased risk of adverse outcomes.^9^ Primary care teams should look beyond patient‐reported memory complaints and prioritize high‐risk clinical profiles (e.g., frailty, multimorbidity, hearing impairment) and informant concerns. In the fragmented, insurance‐based US system, clinical complexity and sensory impairment may delay recognition; embedding brief, validated tools into routine workflows, including the Medicare Annual Wellness Visit, and supporting implementation with electronic health record prompts and staff training may improve detection without overburdening clinicians.^37^ In Brazil's Unified Health System, strengthening primary care capacity through clinician training, improving referral pathways, and expanding access to specialist evaluation may reduce diagnostic gaps, particularly in underserved areas.^5^ However, the higher mortality observed among individuals with undiagnosed dementia does not indicate that case‐finding alone would reduce mortality. This likely reflects a combination of disease severity, frailty, comorbidity burden, and differences in access to and quality of care.^34^, ^35^, ^36^ A better understanding of the mechanisms underlying this association is needed to inform interventions beyond detection, including improved clinical management, patient and family support, and continuity of care.^3^

This study has several strengths. We used harmonized methods in two nationally representative cohorts from high‐ and lower‐income countries to provide population‐based estimates of undiagnosed dementia and its association with 4‐year mortality in the United States and Brazil. Mortality ascertainment relied on validated sources with high completeness, minimizing loss to follow‐up. These cross‐national comparisons clarified the prognostic relevance of undiagnosed dementia and identified characteristics associated with a higher likelihood of undiagnosed dementia across distinct socioeconomic and health‐system contexts.

Some limitations should also be considered. Despite harmonization, differences in measurement and health care context may affect cross‐country comparability. For example, differences in how reported diagnoses were ascertained across cohorts, with broader dementia definitions in the United States and greater emphasis on Alzheimer's disease in Brazil. In addition, our dementia classification relied on validated survey algorithms based on cognitive and functional performance rather than clinical diagnosis, and some misclassification is expected, which would likely attenuate associations.^38^ The cognitive battery did not capture all domains, particularly visuospatial and executive function, and we lacked linkage to administrative health records that could improve diagnostic ascertainment. Finally, self‐ or proxy‐reported dementia diagnosis may reflect limited awareness rather than the absence of a diagnosis, a factor that may itself carry prognostic relevance.^4^, ^39^ Future studies should examine patient‐centered outcomes, including quality of life and functional trajectories, to better characterize the impact of undiagnosed dementia on older adults.

In conclusion, this cross‐national cohort study shows that undiagnosed dementia is common and associated with increased mortality, with a greater burden and pronounced social and healthcare disparities in Brazil compared with the United States. Older adults with undiagnosed dementia had higher mortality than those without dementia in both countries, while mortality was similar for undiagnosed and diagnosed dementia in Brazil. This diagnostic gap identifies a vulnerable group not reached by current diagnostic pathways. As global populations age and the number of people living with dementia increases, these cross‐national differences support efforts to improve timely, equitable detection and access to post‐diagnostic care, particularly in lower‐resource settings.

## AUTHOR CONTRIBUTIONS

**M.J.R.A**.: Funding acquisition; conceptualization; investigation; formal analysis; methodology; writing — original draft; and writing – review and editing.**T.J.A.S**.: Funding acquisition; conceptualization; investigation; formal analysis; methodology; and writing — review and editing. K.E.C.; K.L.: Funding acquisition; conceptualization; investigation; methodology; and writing — review and editing. **S.J.L**.;**L.B**.;**C.P.F**.;**A.C.C.M**.: Conceptualization; methodology; and writing — review and editing.**M.F.L.C**.: Funding acquisition; conceptualization; methodology; and writing — review and editing.**J.V.M.M**.: Data curation; methodology; and writing — review and editing.**A.L.G**.: Conceptualization; investigation; project administration; supervision; methodology; visualization; writing — original draft; and writing — review and editing.**C.K.S**.: Funding acquisition; conceptualization; investigation; supervision; methodology; visualization; writing — original draft; and writing — review and editing.

## CONFLICT OF INTEREST STATEMENT

The authors declare no conflicts of interest. Author disclosures are available in the supporting information

## CONSENT STATEMENT

Informed consent was obtained from all participants prior to enrollment in the US Health and Retirement Study (HRS) and the Brazilian Longitudinal Study of Aging (ELSI‐Brazil).

## Supporting information



Supporting Material: alz71430‐sup‐0001‐SuppMat.docx

Supporting Material: alz71430‐sup‐0002‐SuppMat.pdf

Supporting Material: alz71430‐sup‐0002‐SuppMat.pdf
