# Supplementary material for: Undiagnosed dementia and mortality among older adults in the United States and Brazil: A cross‐national cohort study
Source: Alzheimers Dement. 2026 Apr 29;22(5):e71430. doi: 10.1002/alz.71430 (PMC13128339; doi:10.1002/alz.71430)
Supplement: Supplementary file 1 — Supporting Material: alz71430‐sup‐0001‐SuppMat.docx [file ALZ-22-e71430-s003.docx]

**Undiagnosed dementia and mortality among older adults in the United States and Brazil: a cross-national cohort study**

This supplementary material for online only contains:

**Supplementary Figure S1.** Flowchart of the study sample

**Supplementary Figure S2.** Operationalization of diagnosed and undiagnosed dementia classification

**Supplementary Methods.** Normative sample selection and standardization procedures for defining cognitive impairment

**Supplementary Table S1.** Participants’ characteristics by normative group in the Health and Retirement Study (HRS – United States) and Brazilian Longitudinal Study of Aging (ELSI-Brazil) cohorts

**Supplementary Table S2.** Participant characteristics by cohort: the Health and Retirement Study (HRS – United States) and Brazilian Longitudinal Study of Aging (ELSI-Brazil)

**Supplementary Table S3.** Association between dementia classification status and 4-year mortality among self-respondent participants in the HRS and ELSI-Brazil

**
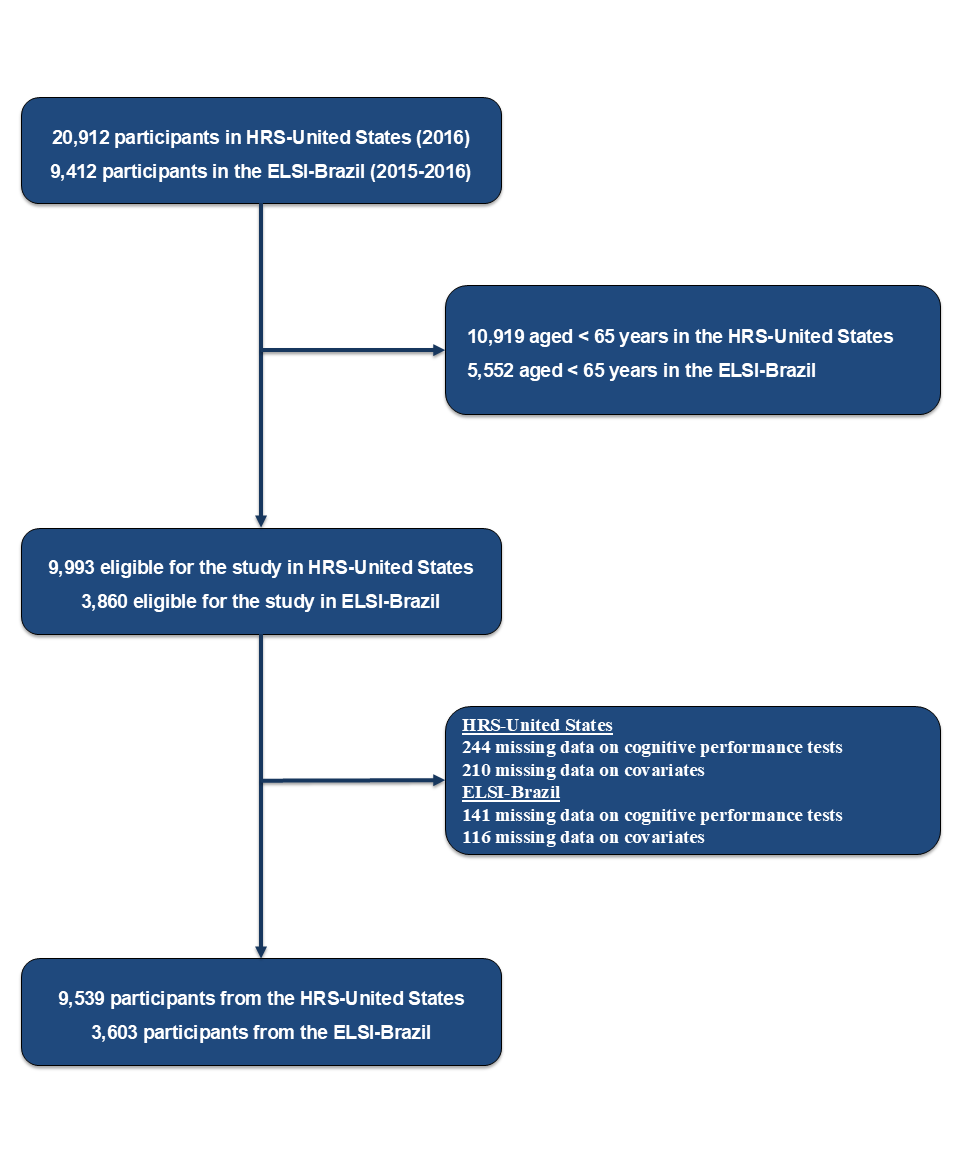
**

**Supplementary Figure S1. Flowchart of the study sample**

HRS = Health and Retirement Study; ELSI-Brazil = Brazilian Longitudinal Study of Aging.


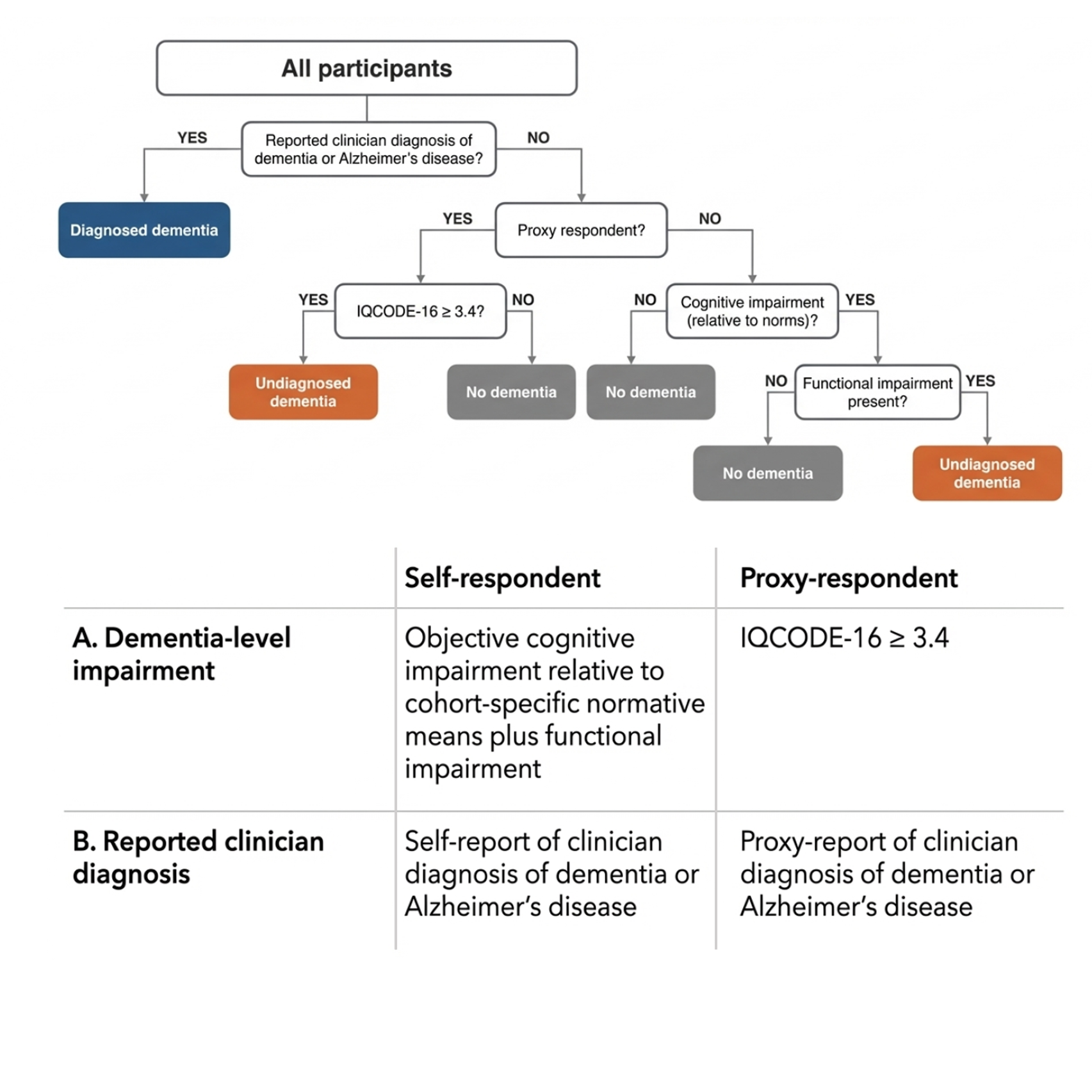


**Supplementary Figure S2. Operationalization of diagnosed and undiagnosed dementia classification**

IQCODE-16 = 16-item Informant Questionnaire on Cognitive Decline in the Elderly.

This flowchart depicts the algorithm used to classify dementia. Diagnosed dementia was defined as self- or proxy-report of a clinician diagnosis of Alzheimer’s disease or dementia. Among participants without a reported diagnosis, dementia classification differed by respondent type. For self-respondents, undiagnosed dementia was defined as cognitive impairment relative to cohort-specific normative means accompanied by functional impairment, defined as difficulty with at least 1 basic activity of daily living (ADL) or at least 2 instrumental activities of daily living (IADL)· For proxy respondents (n = 730 [5·5%]), undiagnosed dementia was defined as an IQCODE-16 score ≥3·4. All other participants were classified as having no dementia.

**Supplementary Methods. Normative sample selection and standardization procedures for defining cognitive impairment**

To identify individuals with cognitive test performance below what would be expected for their age, sex, and education, we constructed cohort-specific normative subsamples using previously established methods from large epidemiologic studies.^1-3^ Participants were excluded from the normative group if they had conditions that could adversely affect cognitive performance and thereby shift normative distributions. Importantly, these exclusions were applied only to the construction of the normative subsample and not to the full analytic sample.

In the Health and Retirement Study (HRS), among 9,539 eligible individuals aged 65 and older, we excluded those with a dementia diagnosis (n = 635), missing cognitive test data across key domains (n = 180; orientation, immediate and delayed recall, verbal fluency, and language), history of stroke (n = 930), other neurological diseases (n = 2), memory complaints (n = 3,300), heavy alcohol consumption (n = 61), a diagnosis of depression (n = 962), other severe psychiatric illnesses (n = 135), or functional limitations in instrumental (IADL; n = 1,031) or basic (ADL; n = 204) activities of daily living. After applying these sequential exclusions, the final normative subsample in HRS comprised 2,099 participants, representing 22·0% of the eligible sample.

Similarly, in the Brazilian Longitudinal Study of Aging (ELSI-Brazil), among 3,603 eligible individuals aged 65 and older, we excluded those with a dementia diagnosis (n = 71), missing cognitive test data across key domains (n = 189; orientation, immediate and delayed recall, verbal fluency, and language), history of stroke (n = 222), other neurological diseases (n = 16), memory complaints (n = 1,702), heavy alcohol consumption (n = 78), a diagnosis of depression (n = 143), other severe psychiatric illnesses (n = 0; none reported), or functional limitations in IADL (n = 345) or ADL (n = 50). After applying these criteria, the final normative subsample in ELSI-Brazil consisted of 787 participants, accounting for 21·8% of the eligible sample.

Within the normative samples of each cohort, we calculated cognitive domain means and standard deviations, stratified by age, sex, and education when appropriate. Cognitive impairment in the full analytic sample was then defined based on performance thresholds relative to the normative subsample: a cognitive deficit was identified when a participant scored at least 1·5 standard deviations below the normative mean in two or more cognitive domains, or when they scored 1·5 standard deviations below the mean in one domain accompanied by at least two other domains scoring 1·0 standard deviation below the mean. This definition is intended to identify cognitive performance substantially below expected levels rather than to assign etiology or clinical diagnosis. This operationalization reflects approaches recommended in prior epidemiologic studies applying robust normative standards to large, aging cohort datasets.^1-3^

This standardized and exclusionary method aimed to minimize biases from comorbidities or functional limitations that could otherwise confound cognitive test results. For example, conditions such as stroke were excluded from the normative sample not because they are unrelated to cognitive impairment, but because they represent established causes of cognitive deficits that could distort normative baselines. It aligns with the strategies applied in previous normative efforts that aimed to establish robust cognitive baselines in population-based studies.^1-3^

**References**

1. Manly JJ, Bell-McGinty S, Tang MX, Schupf N, Stern Y, Mayeux R. Implementing diagnostic criteria and estimating frequency of mild cognitive impairment in an urban community. Arch Neurol. 2005;62(11):1739-46. doi: 10.1001/archneur.62.11.1739.

2. Bertola L, Suemoto CK, Aliberti MJR, et al. Prevalence of Dementia and Cognitive Impairment No Dementia in a Large and Diverse Nationally Representative Sample: The ELSI-Brazil Study. The journals of gerontology Series A, Biological sciences and medical sciences. 2023;78(6):1060-1068. doi: 10.1093/gerona/glad025.

3. Gross AL, Nichols E, Angrisani M, et al. Prevalence of DSM-5 mild and major neurocognitive disorder in India: Results from the LASI-DAD. PLoS One. 2024;19(2):e0297220. doi: 10.1371/journal.pone.0297220.

**Supplementary Table S1. Participants’ characteristics by normative group in the Health and Retirement Study (HRS – United States) and Brazilian Longitudinal Study of Aging (ELSI-Brazil) cohorts**

|  | **HRS – United States** | | | **ELSI-Brazil** | | |
| --- | --- | --- | --- | --- | --- | --- |
|  | **Normative** | **Non-normative** | ***P-***  **value ^a^** | **Normative** | **Non-normative** | ***P-***  **value ^b^** |
| ***Variables*** | **(n = 2,099)** | **(n = 7,440)** |  | **(n = 787)** | **(n = 2,816)** |  |
| ***Cognitive and functional measures*** |  |  |  |  |  |  |
| **Orientation** (0-4), mean (SD) | 3·8 (0·5) | 3·6 (0·7) | <0·001 | 3·6 (0·9) | 3·3 (1·1) | <0·001 |
| **Immediate recall** (0-10), mean (SD) | 5·7 (1·7) | 5·0 (1·7) | <0·001 | 4·2 (1·7) | 3·6 (1·6) | <0·001 |
| **Delayed recall** (0-10), mean (SD) | 4·7 (1·9) | 4·0 (2·0) | <0·001 | 2·7 (1·9) | 2·0 (1·7) | <0·001 |
| **Verbal fluency**, mean (SD) | 18·5 (6·7) | 16·4 (6·8) | <0·001 | 12·2 (4·2) | 10·6 (4·0) | <0·001 |
| **Language** (0-4), mean (SD) | 3·5 (1·1) | 3·2 (1·3) | <0·001 | 2·9 (0·8) | 2·6 (0·9) | <0·001 |
| **ADL limitations (0-6)**, mean (SD) | 0·0 (0·0) | 0·7 (1·4) | <0·001 | 0·0 (0·0) | 0·7 (1·5) | <0·001 |
| **IADL limitations (0-6)**, mean (SD) | 0·0 (0·0) | 0·8 (1·6) | <0·001 | 0·0 (0·0) | 1·3 (1·7) | <0·001 |
| ***Sociodemographic characteristics*** |  |  |  |  |  |  |
| **Age (years)**, mean (SD) | 72·3 (6·8) | 75·4 (7·8) | <0·001 | 72·4 (6·0) | 73·7 (7·2) | <0·001 |
| **Female sex**, % | 48·9 | 58·5 | <0·001 | 51·8 | 59·1 | 0·003 |
| **Race/ethnicity**, % |  |  | 0·38 |  |  | 0·002 |
| **White** | 86·7 | 85·5 |  | 49·6 | 41·1 |  |
| **Black** | 8·6 | 9·6 |  | 42·4 | 51·5 |  |
| **Other** | 4·7 | 4·9 |  | 8·0 | 7·4 |  |
| **Education (years)**, median (IQI) | 13 (12, 16) | 12 (12, 15) | <0·001 | 4 (2, 11) | 3 (0, 4) | <0·001 |
| **Income** (in 1,000 USD), median (IQI)^b^ | 60 (35, 107) | 44 (28, 78) | <0·001 | 9 (6, 14) | 7 (5, 12) | <0·001 |
| **Married or partnered**, % | 65·8 | 53·4 | <0·001 | 55·3 | 53·3 | 0·42 |
| **Rural living**, % | 25·3 | 29·6 | 0·001 | 12·9 | 17·7 | 0·02 |
| ***Health comorbidities*** |  |  |  |  |  |  |
| **Hypertension**, % | 58·0 | 69·7 | <0·001 | 57·8 | 66·2 | 0·01 |
| **Diabetes**, % | 20·2 | 29·8 | <0·001 | 18·3 | 19·5 | 0·57 |
| **Cancer**, % | 16·2 | 21·7 | <0·001 | 6·9 | 8·4 | 0·26 |
| **Lung disease**, % | 4·6 | 14·0 | <0·001 | 3·2 | 7·3 | <0·001 |
| **Stroke**, % | 0·0 | 14·8 | <0·001 | 0·0 | 10·1 | <0·001 |
| **Osteoarthritis**, % | 52·8 | 74·9 | <0·001 | 17·3 | 26·5 | <0·001 |
| **Heart disease**, % | 8·0 | 15·5 | <0·001 | 11·6 | 17·9 | 0·004 |
| **Depression**, % | 0·0 | 30·2 | <0·001 | 0·0 | 21·1 | <0·001 |
| **Multimorbidity** (≥ 2 LTCs), % | 51·2 | 80·4 | <0·001 | 31·8 | 54·3 | <0·001 |
| ***Geriatric conditions*** |  |  |  |  |  |  |
| **Memory complaints**, % | 0·0 | 56·8 | <0·001 | 0·0 | 72·2 | <0·001 |
| **Self-rated poor health**, % | 7·8 | 34·4 | <0·001 | 4·8 | 14·6 | <0·001 |
| **Frail status**, % | 2·0 | 21·1 | <0·001 | 2·5 | 20·0 | <0·001 |
| **Hearing impairment**, % | 11·7 | 29·0 | <0·001 | 12·0 | 31·5 | <0·001 |
| **Visual impairment**, % | 8·6 | 28·2 | <0·001 | 11·1 | 25·9 | <0·001 |
| ***Healthcare access and utilization*** |  |  |  |  |  |  |
| **Having a usual provider**, % | 89·8 | 89·6 | 0·79 | 60·4 | 67·2 | 0·002 |
| **Received specialist care**, % | 76·5 | 72·8 | 0·005 | 58·2 | 56·7 | 0·54 |
| **Recent hospitalization**, % | 18·1 | 33·0 | <0·001 | 6·9 | 13·4 | <0·001 |
| **Out-of-pocket expenditure**, % | 89·8 | 87·1 | 0·008 | 23·0 | 31·4 | <0·001 |

SD = standard deviation; IQI = interquartile interval; LTCs = long-term conditions; ADL limitations = difficulty performing basic activities of daily living (dressing, toileting, bathing, eating, transfers, or walking); IADL limitations = difficulty performing instrumental activities of daily living (managing money, using the telephone, taking medications, shopping, preparing meals, or housekeeping).

All estimates are survey-weighted and account for the complex sampling design of the Health and Retirement Study (HRS) in the United States and the Brazilian Longitudinal Study of Aging (ELSI-Brazil).

^a^ *P*-values represent comparisons between normative and non-normative subsamples within each country.

^b^ Annual household income reported in thousands of US dollars (1,000 USD).

**Supplementary Table S2. Participant characteristics by cohort: the Health and Retirement Study (HRS – United States) and Brazilian Longitudinal Study of Aging (ELSI-Brazil)**

|  | **HRS – United States** | **ELSI-Brazil** |
| --- | --- | --- |
| ***Variables*** | **(n = 9,539)** | **(n = 3,603)** |
| ***Sociodemographic characteristics*** |  |  |
| **Age (years)**, mean (SD) | 74·6 (7·8) | 73·4 (6·9) |
| **Female sex**, % | 56·2 | 57·4 |
| **Race/ethnicity**, % |  |  |
| **White** | 85·8 | 43·1 |
| **Black** | 9·3 | 49·4 |
| **Other** | 4·8 | 7·5 |
| **Education (years)**, median (IQI) | 12 (12, 15) | 3 (0, 5) |
| **Income** (in 1,000 USD), median (IQI)^a^ | 47 (29, 84) | 8 (6, 12) |
| **Married or partnered**, % | 56·4 | 53·8 |
| **Rural living**, % | 28·6 | 16·6 |
| ***Health comorbidities*** |  |  |
| **Hypertension**, % | 66·8 | 64·2 |
| **Diabetes**, % | 27·5 | 19·2 |
| **Cancer**, % | 20·4 | 8·1 |
| **Lung disease**, % | 11·7 | 6·3 |
| **Stroke**, % | 11·2 | 7·8 |
| **Osteoarthritis**, % | 69·5 | 24·3 |
| **Heart disease**, % | 13·7 | 16·5 |
| **Depression**, % | 22·8 | 16·2 |
| **Multimorbidity** (≥ 2 LTCs), % | 73·3 | 49·1 |
| ***Geriatric conditions*** |  |  |
| **Memory complaints**, % | 42·9 | 55·4 |
| **Self-rated poor health**, % | 27·9 | 12·3 |
| **Frail status**, % | 16·4 | 16·0 |
| **Hearing impairment**, % | 24·8 | 27·0 |
| **Visual impairment**, % | 23·4 | 22·5 |
| ***Healthcare access and utilization*** |  |  |
| **Having a usual provider**, % | 89·7 | 65·6 |
| **Received specialist care**, % | 73·7 | 57·1 |
| **Recent hospitalization**, % | 29·4 | 11·9 |
| **Out-of-pocket expenditure**, % | 87·8 | 29·4 |

SD, standard deviation; IQI, interquartile interval; LTCs, long-term conditions.

All estimates are survey-weighted and account for the complex sampling design of the Health and Retirement Study (HRS) in the United States and the Brazilian Longitudinal Study of Aging (ELSI-Brazil).

^a^ Annual household income reported in thousands of US dollars (1,000 USD).

**Supplementary Table S3. Association between dementia classification status and 4-year mortality among self-respondent participants in the HRS and ELSI-Brazil**

|  | **Hazard ratio (95% confidence interval)** | | | |
| --- | --- | --- | --- | --- |
|  | **Model 1: Adjusted for sociodemographic factors** | **Model 1: Undiagnosed vs diagnosed dementia** | **Model 2: Fully adjusted (sociodemographic**  **and clinical factors)** | **Model 2: Undiagnosed**  **vs diagnosed dementia** |
| ***HRS-United States (n=9,036)*** |  |  |  |  |
| **No dementia** | (reference) |  | (reference) |  |
| **Undiagnosed dementia** | 2·72 (2·19–3·38) | (reference) | 2·01 (1·60–2·52) | (reference) |
| **Diagnosed dementia** | 2·14 (1·72–2·68) | 1·27 (0·95–1·69) | 1·75 (1·39–2·21) | 1·15 (0·85–1·55) |
| ***ELSI-Brazil (n=3,376)*** |  |  |  |  |
| **No dementia** | (reference) |  | (reference) |  |
| **Undiagnosed dementia** | 2·11 (1·38–3·23) | (reference) | 1·73 (1·10–2·71) | (reference) |
| **Diagnosed dementia** | 1·12 (0·43–2·94) | 1·88 (0·66–5·33) | 1·00 (0·39–2·52) | 1·74 (0·63–4·84) |

HRS = Health and Retirement Study; ELSI-Brazil = Brazilian Longitudinal Study of Aging; IQCODE-16 = 16-item Informant Questionnaire on Cognitive Decline in the Elderly.

In this analysis, proxy respondents were excluded because cognitive impairment was defined using the IQCODE-16, which requires an informant report (730 [5·5%] overall; 503 [5·3%] in HRS and 227 [6·3%] in ELSI-Brazil)· Estimates were derived from Cox proportional hazards models examining the association between dementia status and time to death over a four-year period· Two models were fitted:

Model 1: adjusted for sociodemographic characteristics (age, sex, race/ethnicity, education, income, marital status, and place of residence);

Model 2: fully adjusted, incorporating sociodemographic and clinical measures (multimorbidity, frailty, and recent hospitalization).

In pooled fully adjusted analyses including a country × undiagnosed dementia interaction term, there was no evidence of heterogeneity by country (interaction HR = 0·80; 95% CI = 0·49–1·30).
