# Supplementary material for: Undiagnosed dementia and mortality among older adults in the United States and Brazil: A cross‐national cohort study
Source: Alzheimers Dement. 2026 Apr 29;22(5):e71430. doi: 10.1002/alz.71430 (PMC13128339; doi:10.1002/alz.71430)
Supplement: Supplementary file 2 — Supporting Material: alz71430‐sup‐0002‐SuppMat.pdf [file ALZ-22-e71430-s002.pdf]

STROBE Statement Checklist: Undiagnosed dementia and mortality among older adults in the United States and Brazil: a cross-national cohort study

	Item	Recommendation	Page No
**Title and abstract**	1	(*a*) Indicate the study's design with a commonly used term in the title or the abstract: *In the abstract, Design, Setting, and Participants subsection: "Population-based cohort study using 2016 data from the US Health and Retirement Study (HRS) and 2015-2016 data from the Brazilian Longitudinal Study of Aging (ELSI-Brazil)."*	2
		(*b*) Provide in the abstract an informative and balanced summary of what was done and what was found: The abstract specifies the data sources, eligibility criteria, exposure definition, outcomes, main models, and key results (proportions and HRs).	2
**Introduction**			
Background/rationale	2	Explain the scientific background and rationale for the investigation being reported The introduction section provides the rationale for the global burden, the magnitude of undiagnosed dementia in previous single-country studies, and why cross-national harmonized comparisons matter.	3
Objectives	3	State specific objectives, including any prespecified hypotheses. *"Using nationally representative cohorts of community-dwelling older adults in the US and Brazil, we estimated the proportion of dementia cases without a reported prior diagnosis, identified sociodemographic, clinical, and health system factors associated with diagnostic gaps, and examined the association between undiagnosed dementia and 4-year mortality."*	4
**Methods**			
Study design	4	Present key elements of study design early in the paper *"This longitudinal observational study used cross-national data from two population-based cohorts, including the US Health and Retirement Study (HRS) and the Brazilian Longitudinal Study of Aging (ELSI-Brazil…"*	4
Setting	5	Describe the setting, locations, and relevant dates, including periods of recruitment, exposure, follow-up, and data collection The methods section provides specific information on the HRS wave (2016) and the ELSI baseline (2015-2016), including cohort procedures for data collection, and the mortality follow-up from 2016 to 2020.	4-5
Participants	6	(*a*) Give the eligibility criteria, and the sources and methods of selection of participants. Describe methods of follow-up Eligibility/exclusions and final analytic samples are provided; follow-up is via cohort-specific mortality sources and censoring at 4 years.	5
		(*b*) For matched studies, give the matching criteria and the number of exposed and unexposed Not applicable.	NA
Variables	7	Clearly define all outcomes, exposures, predictors, potential confounders, and effect modifiers. Give diagnostic criteria, if applicable. The methods section provides the undiagnosed dementia definition, with a detailed description of the applied algorithm in the Supplement; the outcome is 4-year all-cause mortality; covariates were listed by domain (sociodemographic characteristics, comorbidities, geriatric conditions, and healthcare access and utilization). An interaction between undiagnosed dementia and cohorts was tested to assess whether associations with 4-year mortality differed between countries.	5-8 Supplement

Data sources/ measurement	8*	For each variable of interest, give sources of data and details of methods of assessment (measurement). Describe the comparability of assessment methods if there is more than one group.<br>The methods section, with additional information in the Supplement, describes cognitive battery domains, thresholds, functional impairment rules, and IQCODE. There is also information on covariate operationalization and mortality sources. The variables, usual source of care, specialized care, recent hospitalization, and out-of-pocket expenditures, were harmonized and coded as binary indicators (yes/no) to ensure comparability across cohorts.	5-8 Supplement
Bias	9	Describe any efforts to address potential sources of bias<br>The manuscript describes a validated normative subsample approach, harmonized across cohorts, intended to minimize bias in algorithm-based dementia classification. It also uses mortality data from different validated sources in each country. In addition, it presents age- and sex-adjusted cumulative mortality estimates by dementia classification to enhance comparability across cohorts and examines the association between undiagnosed dementia and mortality, adjusted for sociodemographic and clinical confounders.	5-9 Supplement
Study size	10	Explain how the study size was arrived at.<br>"*We restricted both cohorts to participants aged 65 years and older because detailed cognitive assessments in HRS were administered only from age 65 years onward. For comparability, we aligned baseline timing using HRS 2016 and ELSI-Brazil 2015-2016. All 9,993 HRS participants aged 65 years and older and all 3,860 ELSI-Brazil participants aged 65 years and older were eligible for the study. We excluded participants with missing cognitive measures (244 in the US and 141 in Brazil) or covariate data (210 in the US and 116 in Brazil). The final sample included 9,539 HRS participants and 3,603 ELSI-Brazil participants (Supplementary Table S2).*"	5-8 Supplement
Quantitative variables	11	Explain how quantitative variables were handled in the analyses. If applicable, describe which groupings were chosen and why.<br>Prespecified thresholds were applied to define impairment based on cognitive domain z-scores, and functional impairment was used to operationalize dementia classification, consistent with prior studies. Age was modelled continuously, frailty was defined using standard cutoffs, and multimorbidity was defined using standard criteria. Education and income were categorized using within-country quartiles (lowest quartile vs others) to capture relative socioeconomic disadvantage in each setting and enhance comparability across cohorts with different absolute distributions. Usual source of care, specialized care, recent hospitalization, and out-of-pocket expenditures were harmonized and coded as binary indicators (yes/no).	5-8 Supplement
Statistical methods	12	(*a*) Describe all statistical methods, including those used to control for confounding<br>The manuscript specifies survey-weighted descriptive tests and multivariable Poisson and Cox models, and it clearly states the covariate sets used to control confounding (sociodemographic factors and additional clinical measures).	8-9
		(*b*) Describe any methods used to examine subgroups and interactions<br>It describes stratified analyses by country and reports a cohort-by-undiagnosed dementia interaction for mortality.	9
		(*c*) Explain how missing data were addressed.<br>A complete-case analysis was performed due to low missingness (5.1%), as shown in the study flowchart (Supplementary Figure S1).	9
		(d) If applicable, explain how loss to follow-up was addressed.	8-9

		It was handled via standardized time-to-event analyses, including cumulative mortality curves and Cox proportional hazards models.<br>(*e*) Describe any sensitivity analyses.<br>*"In a sensitivity analysis, we restricted the sample to self-respondents to evaluate whether proxy-assessed cognitive impairment, as measured by the IQCODE-16, affected the mortality associations."*	10
**Results**			
Participants	13*	(a) Report numbers of individuals at each stage of study—e.g., numbers potentially eligible, examined for eligibility, confirmed eligible, included in the study, completing follow-up, and analysed<br>All 9,993 HRS participants aged 65 years and older and all 3,860 ELSI-Brazil participants aged 65 years and older were eligible for the study. We excluded participants with missing cognitive measures (244 in the US and 141 in Brazil) or covariate data (210 in the US and 116 in Brazil). The final sample included 9,539 HRS participants and 3,603 ELSI-Brazil participants (Supplementary Figure S1).	5-6<br>Supplement
		(b) Give reasons for non-participation at each stage.<br>The study used two population-based cohorts representing older adults in the US (HRS) and Brazil (ELSI-Brazil). Exclusion from the analytic sample was primarily due to missing cognitive measures or covariate data, resulting in the exclusion of only 5.1% of otherwise eligible participants. All analyses accounted for the complex survey design and sample weights to generate population-based estimates.	5; 9<br>Supplement
		(c) Consider the use of a flow diagram<br>A flowchart of the study sample was provided in the Supplementary Figure S1.	Supplement
Descriptive data	14*	(a) Give characteristics of study participants (e.g., demographic, clinical, social) and information on exposures and potential confounders.<br>The Results report between-cohort participant characteristics (age, race/ethnicity, education, income, comorbidity burden, and health care access and use) in the Supplementary Table S2 and present comparisons by dementia classification (no dementia, undiagnosed dementia, and diagnosed dementia) in Table 1. Characteristics according to inclusion in the normative subsample within each cohort are provided in the Supplement.	10-11<br>Table 1<br>Supplement
		(b) Indicate the number of participants with missing data for each variable of interest.<br>The Results report exclusions due to missing cognitive measures and covariate data, as detailed in the Supplement (study flowchart).	Supplement
		(c) Summarise follow-up time (e.g., average and total amount).<br>The Results report a median follow-up of 4.0 years, and 96.5% had complete follow-up through death or 4 years.	11
Outcome data	15	Report numbers of outcome events or summary measures over time<br>The Results present age- and sex-adjusted cumulative mortality estimates by dementia classification within each country over the 4-year follow-up using time-to-event analysis (Figure 3). Unadjusted mortality estimates were not reported because undiagnosed dementia is strongly associated with age, and crude mortality rates could be misleading. In addition, given the primary aim of enabling cross-national comparisons, unadjusted cumulative mortality would be difficult to interpret across the US and Brazil due to differences in age distributions between populations.	11

Main results	16	(*a*) Give unadjusted estimates and, if applicable, confounder-adjusted estimates and their precision (e.g., 95% confidence interval). Make clear which confounders were adjusted for and why they were included.<br>Table 2 reports unadjusted and adjusted HRs with 95% CIs for dementia groups in each cohort, and the table footnote specifies which sociodemographic and clinical covariates were included in adjusted models. The Results text also reports key adjusted comparisons (including undiagnosed vs diagnosed dementia) with 95% CIs.	11<br>Table 2
		(*b*) Report category boundaries when continuous variables were categorized.<br>Category boundaries (e.g., lowest-quartile thresholds for education and income, and their numeric cut points) are explicitly stated in the Methods section and reiterated in Figure 2 notes.	9<br>Figure 2
		(*c*) If relevant, consider translating estimates of relative risk into absolute risk for a meaningful time period.<br>The Results provide absolute, clinically interpretable measures by reporting age- and sex-adjusted 4-year cumulative mortality for undiagnosed dementia, compared with diagnosed dementia and no dementia, alongside relative measures (HRs).	11<br>Figure 3
Other analyses	17	Report other analyses done—e.g., analyses of subgroups and interactions, and sensitivity analyses.<br>The manuscript reports an interaction test assessing heterogeneity by country and includes a sensitivity analysis restricted to self-respondents to test whether proxy-based cognitive impairment (IQCODE-16) influenced results, with results referenced in the Supplementary Table S3.	11<br>Supplement
**Discussion**			
Key results	18	Summarise key results with reference to study objectives<br>*"This cross-national cohort study indicates that undiagnosed dementia is a substantial and urgent public health problem among older adults in the US and Brazil. Nearly half of older adults with dementia in the US and more than three-quarters in Brazil did not report receiving a formal diagnosis, exposing major gaps in dementia detection across distinct socioeconomic contexts. Undiagnosed dementia was associated with younger age and absence of memory complaints in both countries. In the US, individuals with undiagnosed dementia had less functional impairment than those with diagnosed dementia. In Brazil, undiagnosed dementia was more common among members of underrepresented racial and ethnic groups, those with lower educational attainment, and those living in rural areas. Undiagnosed dementia was associated with higher 4-year mortality than no dementia in the US and with a mortality risk similar to diagnosed dementia in Brazil."*	12
Limitations	19	Discuss limitations of the study, taking into account sources of potential bias or imprecision. Discuss both the direction and the magnitude of any potential bias<br>*"Several limitations should also be considered… differences in measurement and health care context may affect cross-country comparability… some misclassification is expected, which would likely attenuate associations… The cognitive battery did not capture all domains… we lacked linkage to administrative health records… self- or proxy-reported dementia diagnosis may reflect limited awareness…"*	14
Interpretation	20	Give a cautious overall interpretation of results, considering objectives, limitations, multiplicity of analyses, results from similar studies, and other relevant evidence	

		The Discussion provides cautious alternative interpretations for the study findings to the study objectives, weighs key limitations and potential biases, and situates the results within prior cross-national and LMIC evidence on underdiagnosed dementia.<br>*"Few cross-national studies have examined undiagnosed dementia… Prior population-based studies… suggest… 40% to 65%… Estimates from LMICs… 70% to 90%… consistent with our findings… These findings also have prognostic implications…"*<br>*"Alternatively, diagnosis may be more closely linked to access to resources and continuity of care… These findings suggest that estimates based only on diagnosed dementia may underestimate dementia-related mortality."*	12-13
Generalisability	21	Discuss the generalisability (external validity) of the study results<br>The findings are generalizable to community-dwelling adults aged 65 years and older in the US and Brazil, given the use of nationally representative cohorts and survey weights. Results do not extend to institutionalized populations, such as nursing home residents, or to younger adults, who were not included in the analysis. Generalizability to other countries depends on similarities in health system organization and clinical practice.	13-14
		**Other information**	
Funding	22	Give the source of funding and the role of the funders for the present study and, if applicable, for the original study on which the present article is based<br>*"This work was supported by the Alzheimer's Association (23AARFD-1028868) to Drs Aliberti, Avelino-Silva, and Suemoto, by the Health Care Systems Research Network–Older Americans Independence Centers (HCSRN-OAICs) AGING Initiative, funded by the National Institute on Aging (grant R33AG057806), to Drs Lam and Aliberti, and by the Brazilian National Council for Scientific and Technological Development (grant number 421262/2025-7) to Drs Aliberti and Avelino-Silva…"*<br>*"The funders had no role in the design and conduct of the study; collection, management, analysis, and interpretation of the data; preparation, review, or approval of the manuscript; and decision to submit the manuscript for publication."*	20-21
